# Radiosensitizing Effect of Gadolinium Oxide Nanocrystals in NSCLC Cells Under Carbon Ion Irradiation

**DOI:** 10.1186/s11671-019-3152-2

**Published:** 2019-10-21

**Authors:** Feifei Li, Zihou Li, Xiaodong Jin, Yan Liu, Ping Li, Zheyu Shen, Aiguo Wu, Xiaogang Zheng, Weiqiang Chen, Qiang Li

**Affiliations:** 1 Institute of Modern Physics, Chinese Academy of Sciences; Key Laboratory of Heavy Ion Radiation Biology and Medicine of Chinese Academy of Sciences, Key Laboratory of Basic Research on Heavy Ion Radiation Application in Medicine, Lanzhou, 730000 Gansu Province China; 2 University of Chinese Academy of Sciences, Beijing, 100049 China; 3 Key Laboratory of Magnetic Materials and Devices, Chinese Academy of Sciences, Division of Functional Materials and Nano Devices, Ningbo Institute of Materials Technology & Engineering, Chinese Academy of Sciences, Ningbo, 315201 Zhejiang China

**Keywords:** Radiosensitizing effect, Gadolinium oxide nanocrystals, DNA damage, Apoptosis, Cytotoxic autophagy, Carbon ion radiotherapy

## Abstract

**Abstract:**

Gadolinium-based nanomaterials can not only serve as contrast agents but also contribute to sensitization in the radiotherapy of cancers. Among radiotherapies, carbon ion irradiation is considered one of the superior approaches with unique physical and biological advantages. However, only a few metallic nanoparticles have been used to improve carbon ion irradiation. In this study, gadolinium oxide nanocrystals (GONs) were synthesized using a polyol method to decipher the radiosensitizing mechanisms in non-small cell lung cancer (NSCLC) cell lines irradiated by carbon ions. The sensitizer enhancement ratio at the 10% survival level was correlated with the concentration of Gd in NSCLC cells. GONs elicited an increase in hydroxyl radical production in a concentration-dependent manner, and the yield of reactive oxygen species increased obviously in irradiated cells, which led to DNA damage and cell cycle arrest. Apoptosis and cytostatic autophagy were also significantly induced by GONs under carbon ion irradiation. The GONs may serve as an effective theranostic material in carbon ion radiotherapy for NSCLC.

**Graphical Abstract:**

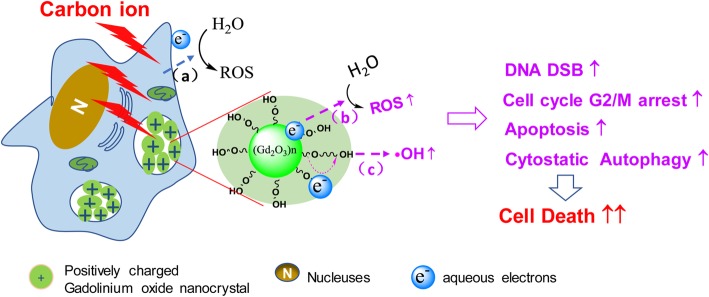

## Introduction

Non-small cell lung cancer (NSCLC) is the most common lung cancer, accounting for approximately 18.4% of the total cancer-related deaths per year and 11.6% of the newly diagnosed cases [1]. However, NSCLC patients are always diagnosed at an advanced phase or with metastasis and therefore are ineligible for surgery and have a poor prognosis; namely, the 5-year survival rate is only 16.1% [2]. Aside from surgery and chemotherapy, radiation therapy is an effective treatment, especially for patients with locally or regionally advanced NSCLC [3]. Currently, more advanced radiotherapy technologies have been developed, such as imaging-guided radiotherapy (IGRT), intensity-modulated radiotherapy (IMRT), and charged-particle therapy (protons and carbon ions), to achieve more precise and sufficient dose delivery to a tumor while sparing the surrounding healthy tissues.

With regard to carbon ions, their physical properties contribute a maximum dose deposition at the end of the particle trajectory followed by a sharp energy fall-off (named the Bragg peak), which permits precise dose delivery to tumors in complex anatomical locations. The use of carbon ions contributes to a higher probability of damage to tumors as well as a lower risk to surrounding health tissue than does conventional radiotherapy [4]. Compared to X-rays, carbon ion irradiation possesses the potential advantages including a better physical dose distribution, a greater reduction in lateral scattering [5], higher relative biological effectiveness (RBE), and a lower oxygen enhancement ratio (OER), all of which are desirable features for killing radioresistant, hypoxic tumors [6]. Consequently, carbon ion therapy is considered one of the superior noninvasive approaches for the treatment of tumors located in highly sensitive tissues such as lung and for tumors that are resistant to conventional radiotherapy [7]. However, a significant dose is administered to healthy tissues in front of the tumor (i.e., at the entrance of the track). It is thus challenging to enhance the biological effect of treatment in the tumor while lowering the dose administered to healthy tissues.

Many efforts have been made to improve the biological effect of heavy ion irradiation, including the use of cellular pathway inhibitors [8], small chemical drugs [9, 10], and metallic nanomaterials [11–13]. Among them, high-Z metal-based nanoparticles possess a high X-ray photon capture cross-section, intensify the production of secondary and Auger electrons, and enhance the reactive oxygen species (ROS). Although they can be used as radiation enhancers for hadron therapy [14, 15], only a few metallic nanoparticles have been used to improve carbon ion irradiation. Kaur found that the presence of glucose-capped gold nanoparticles in HeLa cells led to an enhancement of 41% in the RBE value of carbon ion irradiation [16], and Liu demonstrated that the radiosensitizing effect of gold nanoparticles for carbon ion irradiation was concentration-dependent [11]. Porcel reported that platinum nanoparticles enhanced the DSB damage induced by carbon ion irradiation [12]. In relation to gadolinium-based nanoparticles, only a few studies have been reported concerning carbon ion radiotherapy. Wozny found that AGuIX enhanced the effectiveness of carbon ions to radioresistant head and neck tumor cells [13]. Porcel also found that gadolinium-based nanoparticles (GBNPs) enhanced the sensitivity of Chinese hamster ovary cells to C^6+^ and He^2+^ irradiation [17]. More efforts are needed to expand the utility and to explore the biological mechanisms of metal-based nanoparticles, especially for theranostic reagents such as gadolinium, in carbon ion therapy.

Ultrasmall gadolinium oxide nanocrystals (GONs) have been demonstrated as an advanced T1-weighted magnetic resonance imaging (MRI) contrast due to their high longitudinal relaxivity and small r2/r1 ratios [18, 19]. Our interest has been focused on the radiosensitizing effect and mechanisms of theranostic metal-based nanoparticles for carbon ion irradiation [11, 20]. The aim of this work is to investigate the radiosensitizing effect of GONs on carbon ion irradiation and to unravel the possible mechanisms. Using GONs synthesized by a polyol method, we first evaluated the radiation enhancement of GONs on hydroxyl radical production. After checking the cytotoxicity and cellular uptake, we studied the effect of GONs on the survival fraction of NSCLC cells under carbon ion irradiation using a clonogenic survival assay. Furthermore, we examined cellular ROS production, DNA double-strand breakage (DSB), and cell cycle distribution as well as apoptosis and autophagy induction to unravel the potential mechanisms of the radiosensitizing effect of GONs in NSCLC cells under carbon ion irradiation.

## Results

### Characterization of GONs

**Fig. 1:**
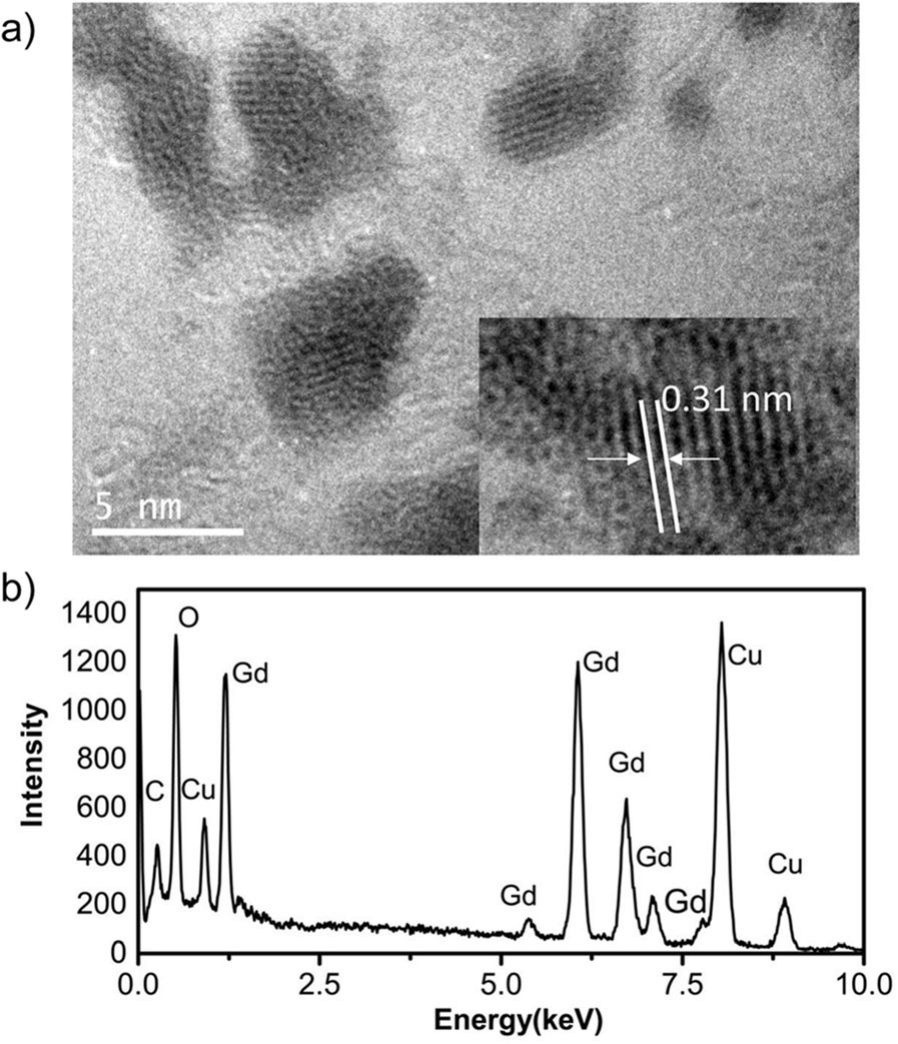
The characteristics of the GONs. **a** HRTEM images of GONs. **b** EDS of GONs

Using a polyol method, the gadolinium oxide nanocrystals (GONs) were synthesized with a size of approximately 2~5 nm (Fig. 1a). As shown in Fig. 1a, the high-resolution transmission electron microscope (HRTEM) image of particles exhibited a regular crystalline lattice with (222) planes (*d* ≈ 3.1 Å). The energy-dispersive X-ray spectra (EDS) of the GONs presented in Fig. 1b indicated the existence of Gd and O in the purified GONs sample. The Cu peak is attributed to the copper grid used for HRTEM. These are consistent with the literature results [19, 21]. In addition, the average hydrodynamic diameter of the GONs was 8.71 ± 2.78 nm (Additional file 1: Figure S1). The synthesized GONs can be concentrated to a stable and clear brownish dispersion and have good compatibility (Additional file 1: Figure S2a).

### GONs Enhance Hydroxyl Radical Production and Cell Damage Under Carbon Ion Irradiation

**Fig. 2:**
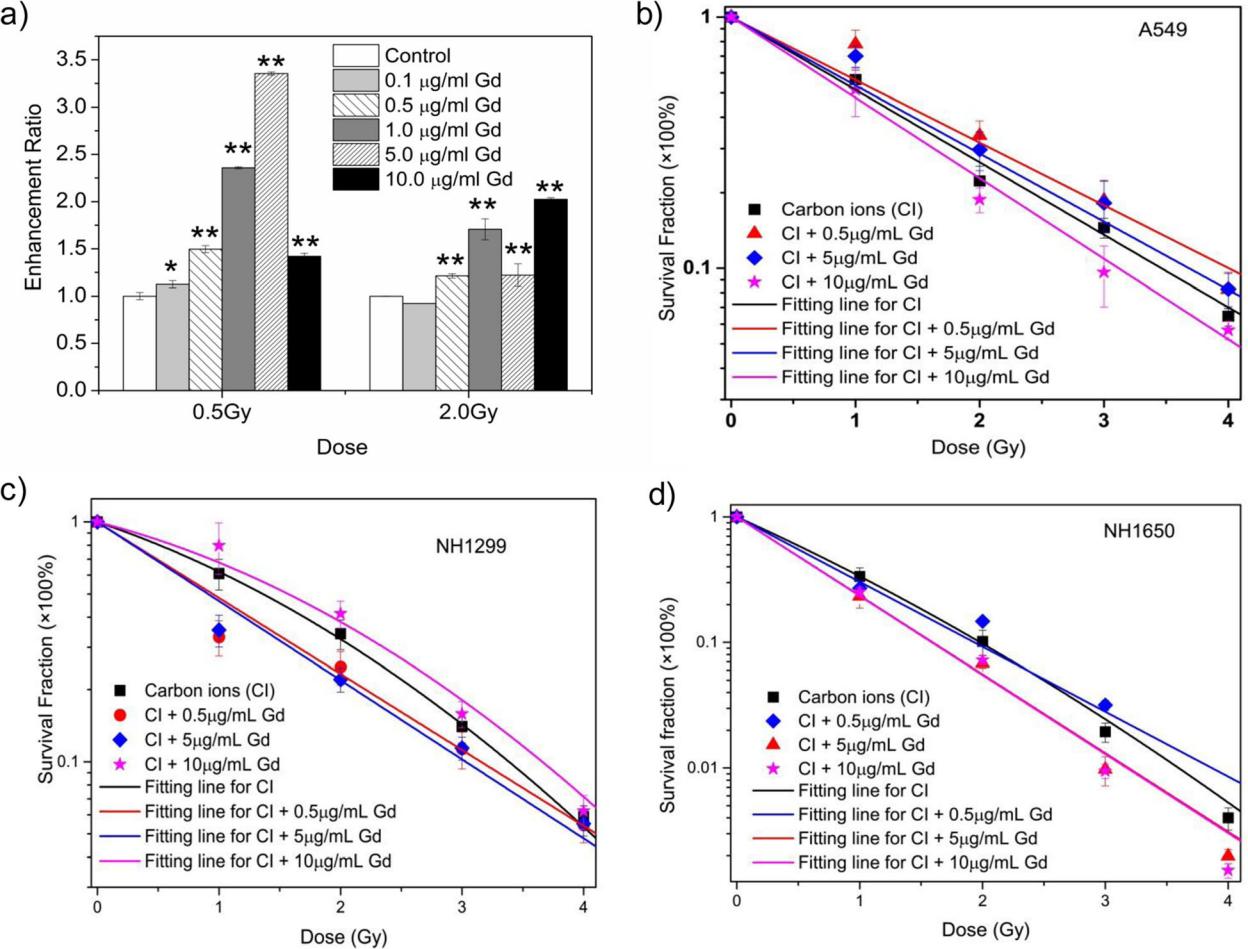
The influence of GONs on hydroxyl radical production and cell survival fractions. **a** The dependence of hydroxyl radical production on the concentration of Gd after carbon ion irradiation. **b–d** The survival curve of GONs-pretreated A549 (**b**), NH1299 (**c**), and NH1650 (**d**) cells under carbon ion irradiation

Carbon ions characteristic of high energy can deposit energy in the medium to produce secondary electrons, which results in water radiolysis and contributes to hydroxyl radical production. We examined the radiation enhancement ratio of GONs on hydroxyl radical production in aqueous solution under carbon ion irradiation using 3-coumarin carboxylic acid (3-CCA) as a probe following the reported procedure [11]. The dependence of the radiation enhancement ratios (ERs) of GONs on the radiation dose is shown in Fig. 2a. The ER for GONs ranged from 1.10~3.32 and 0.93~2.05 for a radiation dose of 0.5 Gy and 2.0 Gy, respectively, and the maximum ER was 3.32 at a Gd concentration of 5.0 μg/mL.

Then, we investigated the survival fractions of the three studied NSCLC cells under carbon ion irradiation with or without GONs, of which the Gd concentration in the medium was 0.5, 5.0, and 10.0 μg/mL. The survival data in each case were fitted with the linear-quadratic model [11]. The clonogenic survival curves are presented in Fig. 2b–d. Compared with carbon ion irradiation alone, the cotreatment caused a more abrupt decrease in survival at a Gd concentration of 10.0 μg/mL in A549 cells and 5.0 μg/mL for NH1299 and NH1650 cells. Using a reported method [11], the sensitizer enhancement ratio (SER) was calculated. As shown in Table 1, the maximum SERs of GONs at the 10% cell survival fraction (SF_10_) were 1.10, 1.11, and 1.20 for A549, NH1299, and NH1650 cells under carbon ion irradiation, respectively. The difference in the SER for the three studied cells might be related to the cellular uptake of GONs (Additional file 1: Figure S2b). These results indicated that GONs can sensitize these three NSCLC cells to carbon ion irradiation in a cell- and concentration-dependent manner, so the optimum radiosensitizing Gd concentrations were used as stated above in our subsequent experiments.

**Table 1:** Summary of fitting parameters, D_SF10_, and SER_SF10_ in the absence or presence of GONs

Carbon-ion irradiation		*α* (Gy^-1^)	*β* (Gy^-2^)	*R* ^2^	D_SF10_ (Gy)	SER_SF10_
A549	Control	0.665		0.993	3.46	
	0.5 μg/mL Gd	0.575		0.996	3.99	0.847
	5.0 μg/mL Gd	0.625		0.96	3.68	0.954
	10.0 μg/mL Gd	0.738		0.996	3.11	1.10
NH1299	Control	0.393	0.084	0.999	3.39	
	0.5 μg/mL Gd	0.73	0	0.95	3.15	1.07
	5.0 μg/mL Gd	0.76	0	0.98	3.01	1.11
	10.0 μg/mL Gd	0.247	0.09	0.987	3.65	0.933
NH1650	Control	1.06	0.073	0.999	1.99	
	0.5 μg/mL Gd	1.24		0.991	1.93	1.03
	5.0 μg/mL Gd	1.44		0.999	1.60	1.20
	10.0 μg/mL Gd	1.45		0.996	1.60	1.20

Coefficients *α*, *β*, and *R*^2^ are fitting parameters using the linear-quadratic model; D_SF10_ means the dosage of carbon ion irradiation at 10% cell survival fraction; SER_SF10_ means sensitizer enhancement ratios of irradiated NSCLC cells at 10% cell fraction

### GONs Reinforce ROS Production During Radiation

**Fig. 3:**
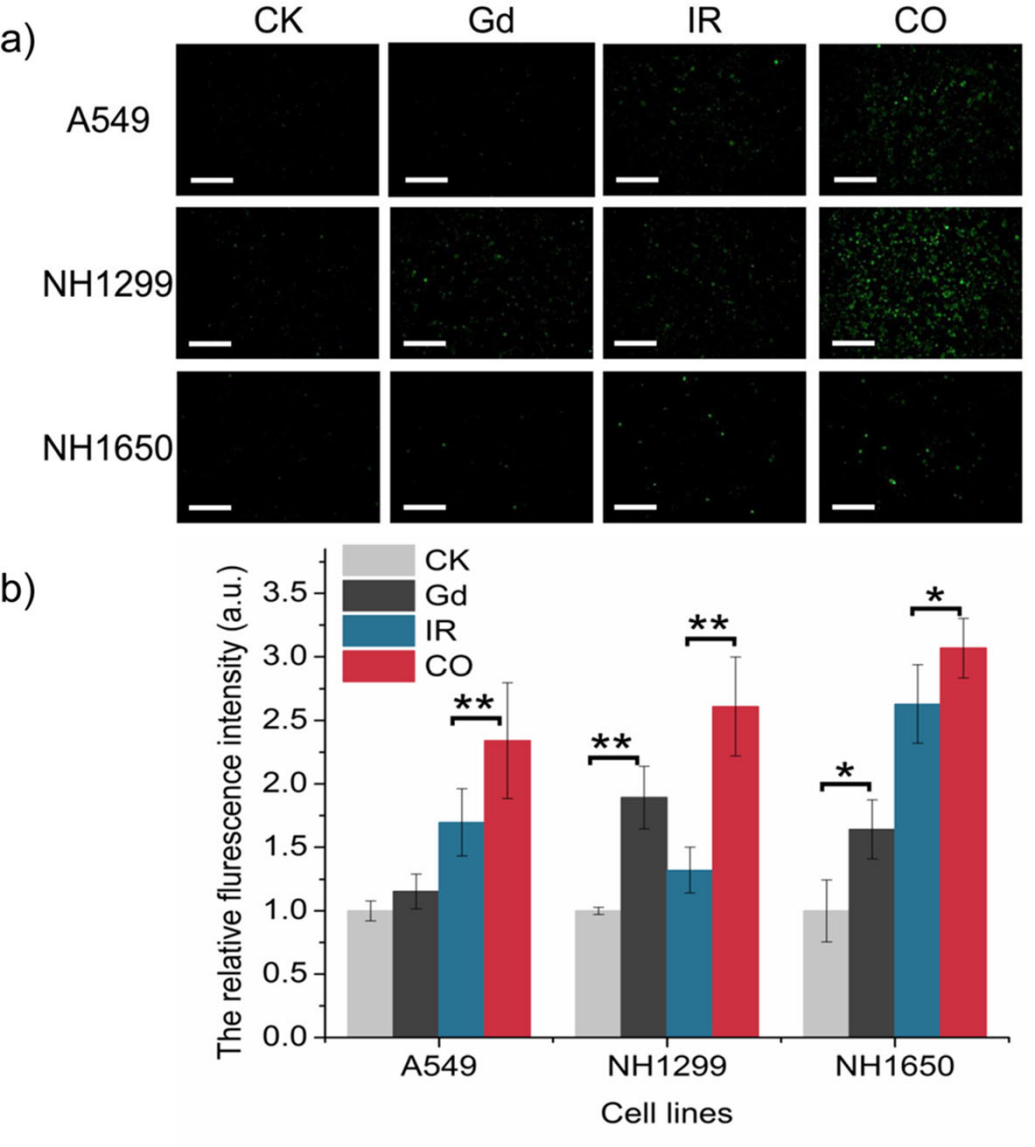
GONs promoted ROS production. **a** The fluorescence images of ROS production observed after radiation by DCFH-DA, “CO” means the cotreatment with GONs and carbon ion radiation; scale bar represents 200 μm. **b** The relative fluorescence intensity of cells with or without GONs was analyzed using ImageJ software. *p < 0.05 or **p < 0.01 represent statistically significant or extremely significant differences, respectively

As mentioned above, many nanomaterials can serve as radiation enhancers because of the increased production of ROS [22, 23]. Therefore, the influence of GONs on the survival fraction of the studied NSCLC cells could be related to the level of reactive oxygen species in vitro. We investigated the ROS levels using the 2,7-dichlorodihydrofluorescein diacetate (DCFH-DA) probe after incubation with GONs. As shown in Fig. 3a and Additional file 1: Figure S3, the cotreatment led to stronger fluorescence emission in all three cells compared to that with carbon ion irradiation alone. In addition, statistical analyses of over 200 cells showed that the relative fluorescence intensities increased 1.16, 1.81, and 1.52 times for A549, NH1299, and NH1650 cells after preincubation with GONs, respectively. Furthermore, the relative fluorescence intensities for A549, NH1299, and NH1650 cells after cotreatment were approximately 1.36-, 2.0-, and 1.19-fold higher than those after radiation alone, indicating that cotreatment significantly enhanced ROS production compared with radiation alone (Fig. 3b). The results indicated that GONs induced an increase in ROS levels in the studied cells exposed to radiation, which may contribute to the radiosensitizing effect stimulated by GONs.

### GONs Strengthen DNA Double-Strand Breaks (DSBs) and Cause Cell Cycle Arrest

**Fig. 4:**
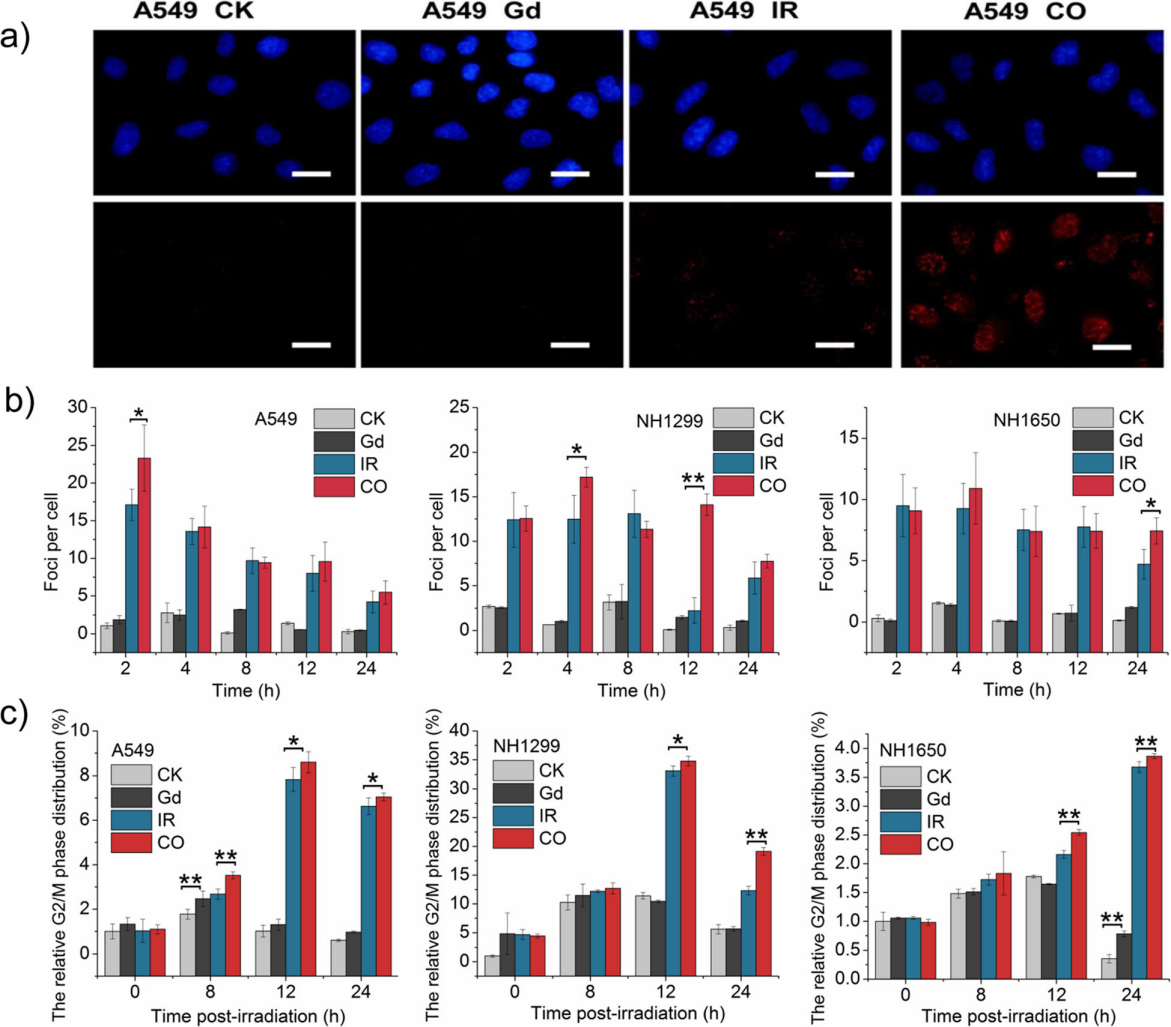
GONs enhanced the number of DNA double-strand breaks and extent of cell cycle arrest. **a** Fluorescent images of γ-H2AX foci in A549 cells were captured with a fluorescence microscope 2 h after radiation; scale bar is 20 μm; the nuclei stained with Hochest33342 are blue; the γ-H2AX foci visualized by incubating with fluorescent antibodies are red. **b** The numbers of foci per cell were counted over at least 50 cells. **c** The relative percentage of cells distributed in G2/M phase of cell cycle progression. *p < 0.05 or **p < 0.01 represent statistically significant or extremely significant differences, respectively

In general, radiation usually leads to nuclear DNA damage, such as DSBs. Phosphorylation of γ-H2AX on Ser139 is considered to be a key marker of DSB [24]. Therefore, the numbers of γ-H2AX foci (red fluorescence point in Fig. 4a) were investigated in the three studied cell lines. The results showed that carbon ion irradiation distinctly increased DNA damage, which was obviously increased in cells pretreated with GONs at several time points after radiation during the dynamic changing process (Fig. 4b).

Cell cycle progression is closely linked to DNA synthesis and damage repair, which activates the cell cycle checkpoints, either allowing enough time for the repair of damaged DNA before moving to the next stage of cell cycle progression or inducing apoptosis in cells carrying unrepaired DNA [25]. As depicted in Fig. 4c and Additional file 1: Figure S4, GONs promoted the relative ratio of cells in G2/M phase in three NSCLC cell lines, especially in NH1650 cells. In addition, GONs treatments increased the level of arrested cells with statistical significance. The time point of cell cycle arrest after radiation varied in three studied cells, which may be related to cell-specific characteristics and to the sequence when the DSBs occurred. For example, serious DSBs caused by cotreatment were observed 2 h post-radiation, and G2/M arrest was detected 8 h after exposure to carbon ions in A549 cell lines, which was earlier than the observed arrest in the other two cell lines. Hence, we deduced that cotreatment with GONs and carbon ion irradiations induced more severe DNA damage and subsequently led to more obvious cell cycle arrest than did radiation alone.

### Apoptosis May Be One of the Radiosensitizing Mechanisms of GONs

**Fig. 5:**
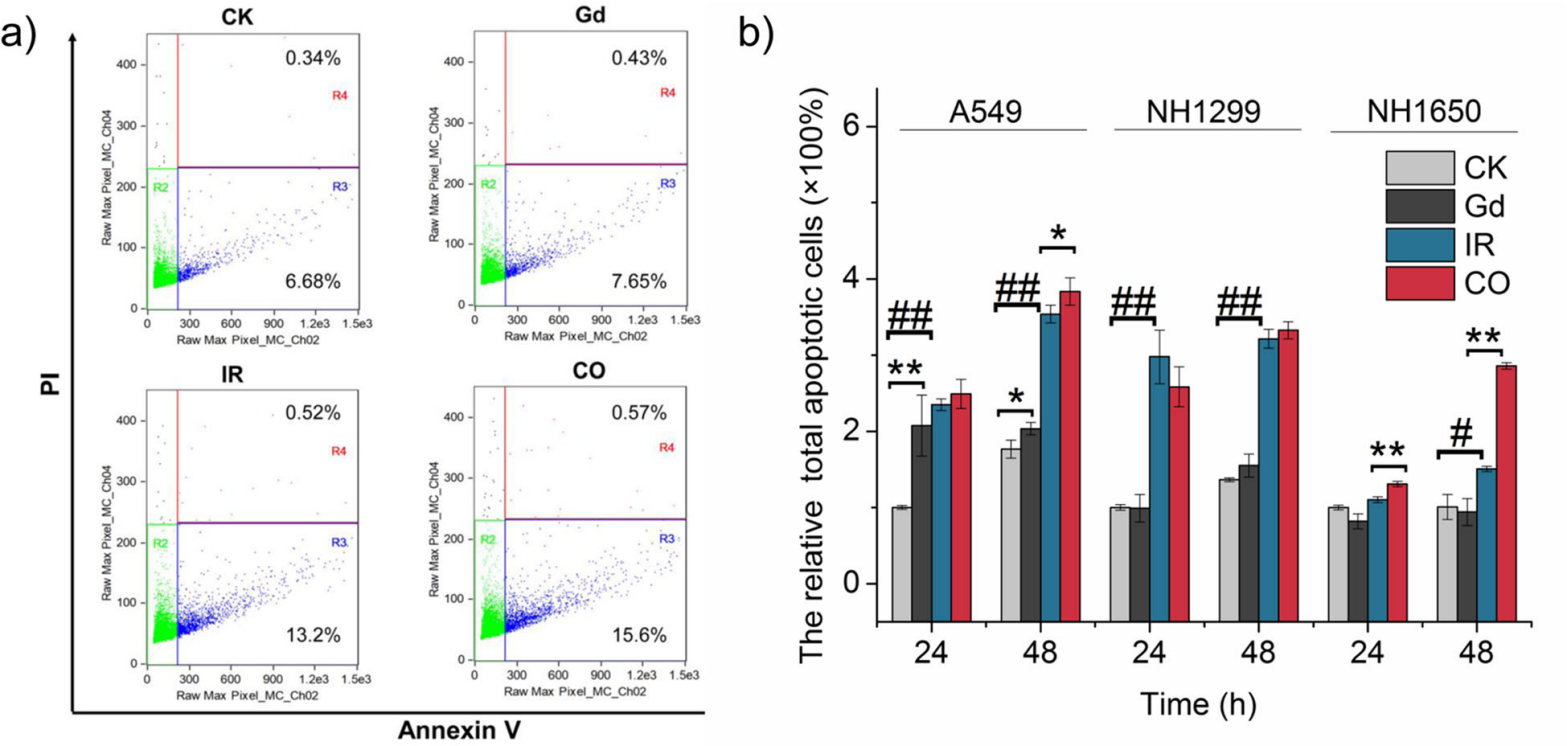
Marked apoptosis was induced by GONs in A549 and NH1650 cells but not in NH1299 cells. **a** The flow cytometry images indicating the apoptotic rates of A549 cells at 48 h after carbon ion radiation. **b** The relative incidence of apoptosis induced by GONs treatment and irradiation. *p < 0.05 or **p < 0.01 represent statistically significant or extremely significant differences, respectively, induced by GONs. Similarly, ^#^*p* < 0.05 or ^##^*p* < 0.01 indicated significant or extremely significant differences, respectively, owing to radiation

To reveal the influence of GONs on the apoptotic rate, an Annexin V-FITC and PI apoptosis kit was used in our study. Figure 5 and Additional file 1: Figure S5 show that cotreatment induced more obvious enhancement in the apoptotic rates than did radiation alone in A549 and NH1650 cells. Although carbon ion radiation elicits obvious apoptosis in NH1299 cells after radiation, there was no significant change in the apoptotic rate of cells preincubated with GONs (Fig. 5b). Moreover, we found that the response to radiation and/or GONs-induced apoptosis was cell-dependent. For instance, GONs induced apoptosis in the NH1650 cell line, and neither carbon ion radiation nor isolated treatment with GONs caused a significant difference in apoptotic GONs induced apoptosis in the NH1650 cell line, and neither carbon ion radiation nor isolated treatment with GONs caused a significant difference in apoptotic incidence at 24 h post treatment; increased the apoptotic rate with extreme significance at both 24 and 48 h after radiation. These results support the notion that apoptosis may be one of the mechanisms responsible for the sensitization of A549 and NH1650 cells to carbon ion radiation.

### GONs Enhanced Cytostatic Autophagy in Response to Carbon Ion Irradiation

**Fig. 6:**
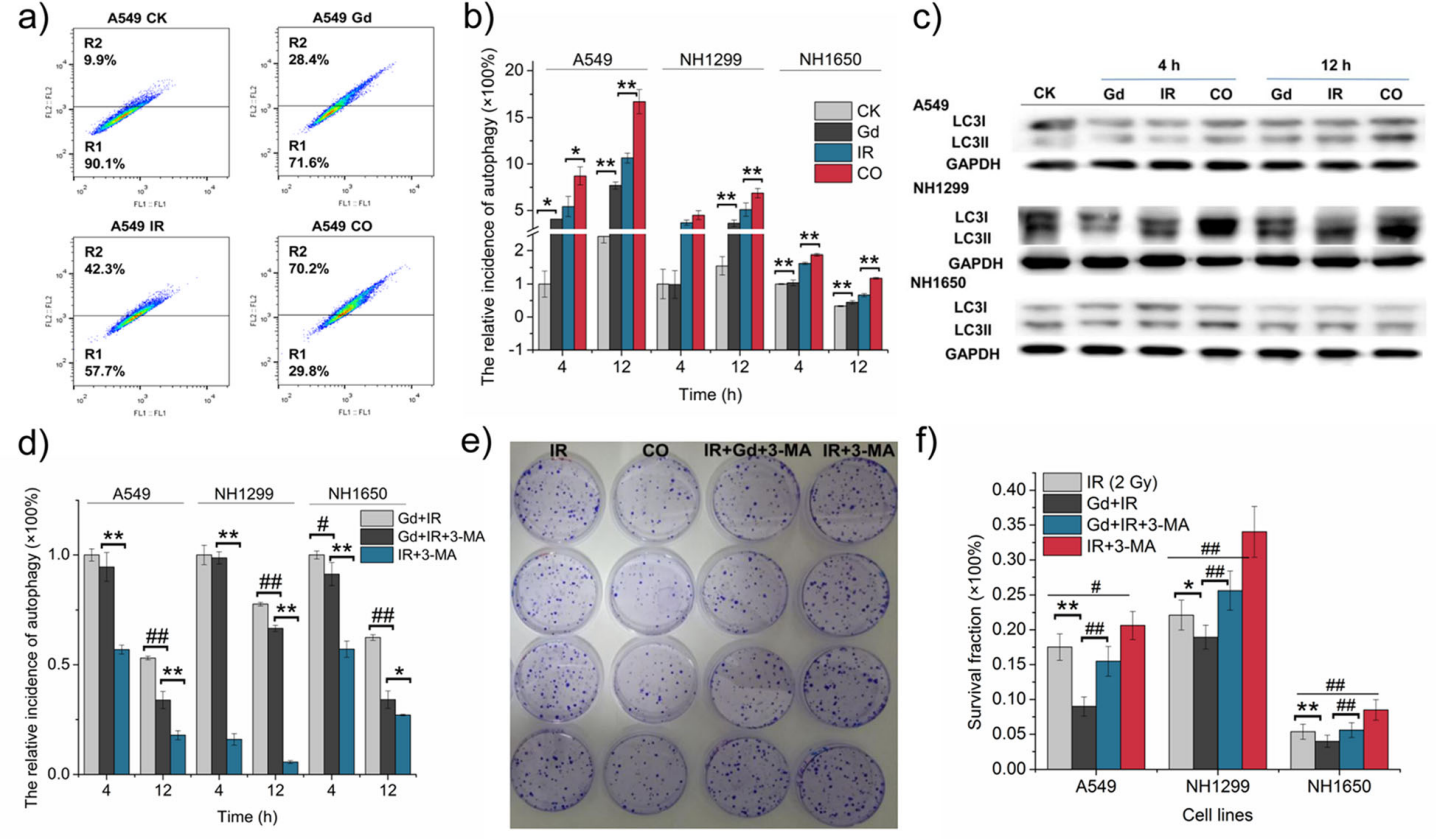
Autophagy-induced death was promoted by GONs in NSCLC cells exposed to carbon ion irradiation. **a** Images of autophagic rates in A549 cells at 12 h post-radiation as measured by flow cytometry (Sysmex CyFlow Cube 6, German). **b** The relative incidence of autophagy in the three studied cell lines. **c** Western blot results of LC3II expression in the three studied cell lines. **d** The relative incidence of GONs-induced autophagy in the presence or absence of 3-MA. **e** The surviving A549 cells were dyed with crystal violet in the clonogenic survival assay. **f** Surviving fraction of NSCLC cells after treatment with 2 Gy carbon ion radiation and/ or GONs with or without 3-MA. *p < 0.05 or **p < 0.01 represent statistically significant or extremely significant differences, respectively, induced by GONs. Similarly, ^#^*p* < 0.05 or ^##^*p* < 0.01 indicated the differences owing to 3-MA treatment

Our previous studies demonstrated that autophagy can be induced by carbon ion radiation in a dose- and LET-dependent manner in various tumor cell lines [26]. To determine whether GONs could induce autophagy and further correlate with the radiosensitivity of the studied cells, we set out to detect the amount of acidic vesicular organelles (AVOs) in the studied NSCLC cells by staining with acridine orange (AO) and using flow cytometry. The flow cytometry images depicted that the autophagic levels of cells preincubated with GONs seemed higher than the levels in the control cells, with significant differences (Fig. 6a, b and Additional file 1: Figure S6a-c). Obviously, cotreatment notably reinforced this tendency in the three NSCLC cell lines compared with radiation alone. Consistent with the flow cytometry results, the expression of LC3-II, the key marker of autophagy, which is the phosphatidylethanolamine (PE)-conjugated form of LC3-I [27], was also enhanced by cotreatment with GONs and carbon ion irradiation in the three studied cell lines (Fig. 6c). Therefore, GONs can promote the occurrence of autophagy in carbon ion-irradiated NSCLC cells.

Although autophagy has elicited extensive attention as a novel target to improve cancer therapy, whether autophagy is responsible for recycling the cellular components and protecting cells from damage or for promoting cell death under severe conditions remains controversial [28, 29]. 3-Methyladenine (3-MA), an inhibitor of autophagic sequestration during the early stage of autophagosome formation [30], was used to elaborate the role of GONs-induced autophagy in irradiated NSCLC cells. By comparing the groups of irradiated cells pretreated with 3-MA with or without GONs (Fig. 6d and Additional file 1: Figure S6d-f), we found that GONs induced a notable enhancement of the autophagic rate. In addition, the flow cytometry results showed that autophagic incidence was significantly reduced in cells incubated with 3-MA (Fig. 6d and Additional file 1: Figure S6d-f). Then, we conducted a clonogenic survival assay (Fig. 6e, f) and found that the survival fraction of cells cotreated with GONs and radiation markedly declined in comparison with cells subjected to radiation alone. However, 3-MA treatment helped to relieve this tendency and promote cell survival. Collectively, these results demonstrate that GONs treatment promoted the radiosensitivity of NSCLC cells to carbon ions through autophagy-induced death.

## Discussion

Currently, more attention has been given to metal-based nanomaterials that serve as either radiosensitizers or synergistic cell-killing effectors in a diverse number of tumor cells [20, 31]. Regarding the study of GBNPs, most of them have been used as MRI contrast agents [32–34]. Recently, there have been efforts to study the radiosensitizing properties of GBNPs, opening the door for GBNPs to be used as potential theranostic agents [35, 36], while the biological radiosensitizing mechanisms of GONs under carbon ion irradiation still need further study.

In this study, we observed the radiosensitizing effect of GONs in NSCLC cells by clonogenic assay, which was related to the Gd concentration in A549 and NH1650 cells; namely, a lower Gd concentration in the medium had no effect, while an obvious radiosensitizing effect of GONs was detected with an increase in the Gd concentration. However, in NH1299 cells, the higher Gd concentration (10.0 μg/mL) showed a protective effect, which may be due to the hydroxyl radical quenching caused by higher Gd cell uptake (Additional file 1: Figure S2b). The difference in cell uptake of GONs might stem from the cells’ essential status [37]. In addition, the production of hydroxyl radicals and ROS increased significantly after incubation with GONs in our study, which was consistent with our perception that Gd, one of the high-Z numbered metal-based nanoparticles, can intensify the production of secondary or Auger electrons and enhance ROS production through the radiolysis of water molecules [14, 15, 38]. Similarly, Seo also found that core–inner-valence ionization of GONs can deexcite electrons via potent Gd-Gd interatomic deexcitation processes, which contributed to the enhancement of GONs in the production of ROS under photon and proton irradiation [19]. We hypothesized that the increased ROS may further trigger catalyzing oxidation reactions [39].

It has been demonstrated without doubt that severe ROS may lead to DNA damage because of its active unpaired electron [38]. DNA damage can mainly be divided into endogenous damages caused by ROS produced from normal metabolism and exogenous damage induced by ionization, manmade chemical materials, and other external stresses [40]. In this study, obvious DSB induction was observed after cotreatment with GONs and carbon ion irradiation compared with that observed with irradiation alone, as shown in Fig. 4a. Consistent with our findings, AGuIX gadolinium nanoparticles also intensified DSB occurrence in head and neck tumor cells exposed to carbon ion irradiation [13]. In contrast, Gd-, Pt-, and Au-based nanoparticles approximately 2–3 nm in size all made no difference to the DSB occurrence under γ-rays, as newly reported by Pagáčová [41]. In our previous study, we also found that cotreatment with GONs and X-ray irradiation failed to induce more serious DSBs than that observed with irradiation alone [42]. The differences may be attributed to the predominance of carbon ion beams and the high Z property of Gd, namely, in contrast with conventional X-rays or γ-rays, carbon ion irradiation can instinctively lead to complex DSBs that are difficult to repair [43, 44]. Moreover, similar to Porcel’s study [12], Gd could emit showers of secondary and Auger electrons, as well as ROS under carbon ion irradiation, and finally amplifies the efficiency of severe DNA damage. When faced with serious DNA damage, cells often initiate DNA damage responses, such as cell cycle arrest and DNA repair [45, 46]. In our study, cell cycle arrest was also found after GONs treatment besides DSBs, which is consistent with Kansara’s results [47]. After evolving signaling cascades, cells with repaired DNA could resume normal cell cycle progression [45]. Once the lesions of DNA were beyond repair, cells executed cell death programs such as apoptosis [48].

In depth, apoptosis was induced in the three studied cell lines in our work; moreover, the apoptotic incidence significantly increased with GONs treatment in A549 and NH1650 cells. With regard to NH1299 cells, which has a deletion of the p53 gene, apoptosis can be induced by carbon ion irradiation, and GONs made no contribution in promoting this tendency. Hence, we hypothesized that carbon ion radiation induced p53-independent apoptosis in NH1299 cells. Emerging studies showed that p53 may be one, but not the sole element that correlated with apoptosis in NSCLC. Similarly, the specific BCL-2 inhibitor ABT-263 can enhance cisplatin-induced apoptosis regardless of the presence or absence of p53 in NSCLC cells [49]. Genistein also induced p53-independent apoptosis in NSCLC cell lines [50]. Similar with the results of cellular studies, Lai et al. found that the expression of p53 had nothing to do with pathological staging and had no correlation with the prognosis of NSCLC by investigating 114 NSCLC cases with different clinical stages [51]. Moreover, p21, which can be activated in a p53-dependent or p53-independent manner, plays a pivotal role in the regulation of the cell cycle and apoptosis after DNA damage [52]. Collectively, p53 may not contribute to apoptosis as a decisive factor in carbon ion-irradiated NSCLC cell lines. Furthermore, other mechanisms may contribute to the radiosensitization of GONs in NH1299 cells.

In addition to apoptosis, autophagy may also be elicited when DNA damage occurs. Autophagy plays a role in DNA repair as a double-edged sword. Autophagy can regulate some of the proteins involved in DNA repair and can also be specifically initiated by several DNA repair molecules. Autophagy may function as a cell death program to eliminate abnormal cells with irreparable DNA damage [53]. Studies have demonstrated that gold [54], silver [55], zinc [56], titanium [57], and other metal-based nanoparticles can induce autophagy. Consistent with these studies, GONs induced autophagy in our studied NSCLC cells as well. Hence, we hypothesize that autophagy, but not apoptosis, may mainly account for the radiosensitizing effect of GONs in NH1299 cells. We used the autophagy inhibitor 3-MA to explore whether GON-induced autophagy plays a pro-survival role or acts as a cell death mechanism. As shown in Fig. 6d and Additional file 1: Figure S6d-f, the results of flow cytometry indicated that autophagy was obviously inhibited by 3-MA; then, autophagy-induced cell death was verified by a clonogenic survival assay. Consequently, cytostatic autophagy played an important role in sensitizing NSCLC cells pretreated with GONs under carbon ion irradiation.

## Conclusions

In summary, pretreatment with GONs led to the enhancement of hydroxyl radical and ROS production, which contributes to cell cycle arrest at G2/M phase to allow for repair of damaged DNA with DSBs. Then, apoptosis and cytostatic autophagy were induced to relieve severe DNA damage and finally sensitize NSCLC cell lines to carbon ion radiation. Moreover, although both autophagy and apoptosis were elicited in the studied NSCLC cells, cytostatic autophagy induced by GONs may play a pivotal role in NH1299 cells. Based on the good biocompatibility, the instinctive advantage of Gd as an MRI contrast agent, and the sensitization effect stated above, GONs may be a potential theranostic sensitizer in NSCLC patients under carbon ion radiotherapy after further in vivo preclinical studies.

Although the physical and chemical mechanisms concerning GBNP-mediated radiotherapy have been well elaborated, the clinical applications of GBNPs remain challenging due to the shortage of specific biomechanisms. In future work, high-throughput proteomic methods based on liquid chromatography-mass spectrometry will be used to decipher the specific biomarkers that respond to GONs treatment and regulate radiosensitivity under carbon ion irradiation. In addition, it is well known that the radiosensitizing effect of GONs is related to its surface modification as well as to the size of the core. Therefore, whether enhanced radiosensitivity toward carbon ion irradiation is induced by other types of GONs will also be examined in our future work.

## Materials and Methods

### Synthesis and Characterization of GONs

The synthesis and characterization of GONs are detailed in our previous study using a reported polyol method [19]. Approximately 1.0 μL of diluted GONs was dropped onto a copper grid and dried at room temperature before observation. The HRTEM images, as well as EDS data, were performed on a JEOL-2100 (JEOL, Japan) instrument with an operating voltage of 200 kV.

### Cell Culture

The human NSCLC cell lines A549, NH1299, and NH1650 were purchased from the Type Culture Collection of the Chinese Academy of Sciences (Shanghai, China). These three well-known NSCLC cells were grown in RPMI 1640 medium (Thermo Fisher Scientific Inc., Waltham, MA) with 10% heat-inactivated fetal bovine serum (FBS; Bailing Bio, Lanzhou, China) at 37 °C in a humidified 5% CO_2_ atmosphere. 3-MA (Selleck.cn, USA) was used as an inhibitor of autophagy 4 h before radiation.

### Carbon Ion Irradiation

Cells were plated into 35-mm Petri dishes overnight and then preincubated with GONs, of which the specific concentrations of Gd were 0.5, 5.0, and 10.0 μg/mL in the medium. After 24 h of treatment with GONs, cells were subjected to carbon ion irradiation at the heavy ion research facility in Lanzhou (HIRFL) at room temperature. The energy of the carbon ion was 100 MeV/u with a linear energy transfer (LET) of 50 keV/μm. The dose rate was approximately 2.0 Gy/min.

### Measurement of Hydroxyl Radical Production

Hydroxyl radical production was evaluated using 3-CCA (J&K Chemical Co. Ltd., China) as a probe. The solution of 3-CCA was prepared following the reported procedure [58]. The diluted solutions, of which the Gd concentrations were 0, 0.1, 0.5, 1.0, 5.0, and 10.0 μg/mL, were equally added into the wells of 96-well black-bottom plates with or without radiation and measured at an excitation wavelength at 395 nm and emission wavelength at 442 nm with a microplate reader (Infinite F200/M200, TECAN Co., Switzerland) protected from light.

### Clonogenic Survival Assay

After being irradiated with 0, 1, 2, 3, and 4 Gy carbon ions, the trypsin-dispersed cell suspensions were counted, diluted, and finally seeded into Φ60 dishes in 5 mL of complete media. Then, colonies were stained with crystal violet for 30 min and carefully washed after 14 days of incubation. Colonies with more than 50 cells were recorded as survivors and counted manually under an inverted microscope. Measured survival data were fitted using the linear-quadratic (LQ) model.

### Reactive Oxygen Species (ROS) Detection

ROS were evaluated using DCFH-DA (Solarbio Life Sciences, China), which is a fluorogenic dye that measures hydroxyl, peroxyl, and other ROS activities within the cells [59]. Cells with or without preincubation of GONs were treated with DCFH-DA in serum-free medium and then exposed to 2.0 Gy carbon ion irradiation. After coincubation with DCFH-DA for 30 min at 37 °C, the fluorescence of the cells was detected with a fluorescence microscope (Olympus Optical Co., Japan). Afterward, the mean cellular fluorescence was calculated using ImageJ software by analyzing more than 200 cells.

### Immunofluorescence Assay

To explore whether cotreatment with GONs and carbon ion radiation enhanced the number of DNA double-strand breaks (DSBs), γ-H2AX, one of the key factors in the DNA damage response [48], was used at 4 °C overnight in our study after cells were fixed with 4% paraformaldehyde, permeabilized with 0.3% Triton X-100, and blocked with 5% BSA. Then, the cells were washed and incubated with donkey anti-mouse secondary antibody for 1.5 h at room temperature. Before the foci were observed with a fluorescence microscope (Olympus Optical Co., Japan), Hoechst 33342 was used for staining the nuclei. The mean values of the foci were counted based on the presence of at least 50 cells.

### Flow Cytometry

The influence of GONs and/or 2 Gy carbon ion irradiation on cell cycle progression was analyzed with flow cytometry (Sysmex CyFlow Cube 6, German). After being harvested and fixed in 70% ice-cold ethanol at − 20 °C for at least 48 h, the cells were stained with PBS containing 100 μg/mL RNase, 0.2% Triton X100, and 50 μg/mL propidium iodide (PI; Sigma-Aldrich Co., US) for 20 min on ice. Next, apoptosis was detected with flow cytometry using an Annexin V-FITC and PI apoptosis kit (Roche Diagnostics, Indianapolis, IN) according to the manufacturer’s protocol. With regard to autophagy incidence, cells were incubated with a 1.0 μg/mL solution of acridine orange (AO) for 15 min, washed, collected, and then quantified by flow cytometry at the indicated time points after irradiation. The contribution of GONs to the relative autophagic incidence of irradiated cells pretreated with 3-MA was also measured by flow cytometry (Amnis, Seattle, WA).

### Western Blot Analysis

Cellular extracts were obtained after they were washed with PBS twice and treated with cell lysis buffers. After sonication at 3 s/8 s intervals for a total of 3 min, centrifugation at 12,000 rpm for 20 min at 4 °C, and then quantitative determination of the concentrations by the Bradford assay, the proteins were separated by SDS–PAGE and transferred to PVDF membranes. Blots were incubated with the LC3-II and GAPDH primary antibodies (all purchased from Cell Signaling Technology®, Danvers, MA) and the corresponding secondary antibody and visualized by enhanced chemiluminescence.

### Statistical Analysis

Quantitative data of independent trials repeated three times are expressed as the mean ± standard deviation (SD). Comparisons of the data derived from the controls and treatments were performed using one-way ANOVA with SPSS v. 16.0 (SPSS/IBM Corp., Armonk, NY). Differences were considered statistically significant and statistically extremely significant when *p* < 0.05 and *p* < 0.01, respectively.

## Supplementary information

**Additional file 1: Figure S1.** The hydrodynamic diameter of GONs was detected by dynamic light scattering. **Figure S2.** Cytotoxicity and cellular uptake of GONs. **Figure S3.** Original images of the relative cellular fluorescence intensity analyzed using ImageJ software. **Figure S4.** Flow cytometry images of cell cycle distribution in the three studied cell lines at 24 h after radiation. **Figure S5.** Flow cytometry images of the apoptotic rates. a The fluorescence images of single A549 cell detected by Amnis flow cytometry. b, c Flow cytometry images of the apoptotic rates of NH1299 (b) and NH1650 (c) cells at 48 h after carbon ion radiation. **Figure S6.** Flow cytometry images of autophagic rates at 12 h after radiation in A549 (a, d), NH1299 (b, e) and NH1650 (c, f) cells subjected to various treatments in the absence or presence of 3-MA.

## Data Availability

Not applicable

## Abbreviations

3-CCA: Coumarin-3-carboxylic acid
3-MA: 3-Methyladenine
DCFH-DA: Dichlorodihydrofluorescein diacetate
EDS: Energy dispersive X-ray spectra
GBNPs: Gadolinium-based nanoparticles
GONs: Gadolinium oxide nanoparticles
HRTEM: High-resolution transmission electron microscope
IGRT: Imaging-guided radiotherapy
IMRT: Intensity-modulated radiotherapy
MRI: Magnetic resonance imaging
NSCLC: Non-small cell lung cancer
OER: Oxygen enhancement ratio
PI: Propidium iodide
ROS: Reactive oxygen species
SER: Sensitizer enhancement ratios
