# Supplementary material for: Radiosensitizing Effect of Gadolinium Oxide Nanocrystals in NSCLC Cells Under Carbon Ion Irradiation
Source: Nanoscale Res Lett. 2019 Oct 21;14:328. doi: 10.1186/s11671-019-3152-2 (PMC6803611; doi:10.1186/s11671-019-3152-2)
Supplement: Supplementary file 1 — Additional file 1: Figure S1. The hydrodynamic diameter of GONs was detected by dynamic light scattering. Figure S2. Cytotoxicity and cellular uptake of GONs. Figure S3. Original images of the relative cellular fluorescence intensity analyzed using ImageJ software. Figure S4. Flow cytometry images of cell cycle distribution in the three studied cell lines at 24 h after radiation. Figure S5. Flow cytometry images of the apoptotic rates. a The fluorescence images of single A549 cell detected by Amnis flow cytometry. b, c Flow cytometry images of the apoptotic rates of NH1299 (b) and NH1650 (c) cells at 48 h after carbon ion radiation. Figure S6. Flow cytometry images of autophagic rates at 12 h after radiation in A549 (a, d), NH1299 (b, e) and NH1650 (c, f) cells subjected to various treatments in the absence or presence of 3-MA. [file 11671_2019_3152_MOESM1_ESM.doc]

**Radiosensitizing Effect of Gadolinium Oxide Nanocrystals in NSCLC Cells under Carbon Ion Irradiation**

Feifei Li ^1, 2^, Zihou Li ^2, 3^, Xiaodong Jin ^1^, Yan Liu ^1^, Ping Li ^1^, Zheyu Shen ^3^, Aiguo Wu ^3^, Xiaogang Zheng^1^, Weiqiang Chen ^1^,*and Qiang Li ^1^,*

1. Institute of Modern Physics, Chinese Academy of Sciences; Key Laboratory of Heavy Ion Radiation Biology and Medicine of Chinese Academy of Sciences, Key Laboratory of Basic Research on Heavy Ion Radiation Application in Medicine, Gansu Province, Lanzhou 730000, China
2. University of Chinese Academy of Sciences, Beijing 100049, China
3. Key Laboratory of Magnetic Materials and Devices, Chinese Academy of Sciences, Division of Functional Materials and Nano Devices, Ningbo Institute of Materials Technology & Engineering, Chinese Academy of Sciences, Ningbo, Zhejiang 315201, China

***** Correspondence: [chenwq7315@impcas.ac.cn](mailto:chenwq7315@impcas.ac.cn); [liqiang@impcas.ac.cn](mailto:liqiang@impcas.ac.cn)

**Methods**

**The synthesis of gadolinium oxide nanocrstals (GONs)**

GONs were synthesized following a previously reported method [1]. Briefly, 2.733g (6.0 mmol) Gadolinium (III) nitrate hexahydrate (Gd (NO_3_)_3_·6H_2_O, 99%) was dissolved in 26 mL diethylene glycol (DEG, 99%). The solution was heated to 100^o^C with vigorous stirring. Then, a sodium hydroxide (NaOH, 99%) solution in DEG at a concentration of 10 mg/mL, was added into the above solution drop by drop slowly. After that, the temperature of the resulting mixture was raised to 140^o^C for 1.0 h, then 175^o^C for another 4.0 h. The obtained GONs sample was naturally cooled, transferred into a member with cut-off of 3500 Da, purified by membrane dialysis with ultrapure water (18.25 MΩ, Milli-Q) for 72 h. The water was replaced every 8 h. After dialysis, the resulting GONs solution was concentrated. The reagents were all purchased from Aladdin Industrial Inc. (Shanghai, China).

**Dynamic light scattering (DLS) measurement**

The hydrodynamic diameter of GONs was measured by DLS method on a Zeta particle size analyzer (Nano-ZS, Malvern, England) at room temperature. The data were collected on an autocorrelator with a detection angle for the scattered light of 173 ^o^.

**Cell viability**

To detect the viability of cells pretreated with GONs, a CCK-8 kit was used for sensitive colorimetric assays for the determination of proliferation and cytotoxicity. Initially, approximately 10,000 cells per well were plated into 96-well plates and cultured in 200 μL RPMI 1640 medium (Thermo Fisher Scientific Inc., Waltham, MA, USA) at 37^o^C overnight to adhere. Then, cells were co-incubated in medium with multiple concentrations of GONs (at Gd concentration of 0, 0.5, 1, 5, 10, 50 100, 200 μg/mL) at 37^o^C for 24 h. Afterwards, cells were washed with PBS twice, incubated in medium containing CCK-8 kit (Apexbio, China) while protected from light, and then detected at a wavelength of 450 nm by a microplate reader (Thermo Fisher Scientific Inc.) within 4 hours. The relative cell viability was calculated by the absorbances normalized to the signal intensity of cells without GONs treatment.

**Cellular Gadolinium concentration measurement**

The cellular Gd concentration was measured by inductively coupled plasma atomic emission spectrometry (ICP-AES) on an IRIS ER/S (Thermo Jarrell Ash, USA) using external calibration method. To obtain cellular Gd concentrations, the cells were pretreated with various concentrations (0.5, 5.0, and 10.0 μg/mL) of Gd for 24 hours. The cells were harvested, counted, and dried, and then resuspended and ionized in 1.0 mL of aqua regia. After measurement, the cellular Gd mass was calculated by dividing the Gd content by the total cell number.

**Calculation of Enhancement ratio of GONs**

We examined the radiation enhancement ratio of the GONs on hydroxyl radical production in aqueous solutions exposed to carbon ions using 3-CCA as a probe following the reported procedure [2]. Firstly, we checked the correlation between the fluorescence intensity of 3-CCA solution and the dose of carbon ion irradiation. Then we measured the change of fluorescence intensity in absence or presence of GONs with carbon-ion irradiation. Enhancement ratio (ER) at certain dose was defined as equation 1.

ER = F_i_/F_0_ (1)

Where ER is the enhancement factor of GONs concerning hydroxyl radical production at each dose; F_i_ and F_0_ are the fluorescence intensity variations of 3-CCA probe solutions with or without GONs under carbon ion irradiation at the same dose, respectively.

**The clonogenic survival assay analysis using linear-quadratic model**

The cell survival data were simulated with the linear-quadratic model (Equation 2) [3].


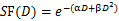
 (2)

Where SF is the survival fraction, D is the radiation dose, α and β are the coefficients of the linear and quadratic terms, respectively.

The sensitizer enhancement ratios (SER) of GONs at certain concentrations were calculated by the ratio of the required dose without GONs to the dose with GONs at the 10% survival level for carbon ions irradiation.

**Supplementary figure legends**

**Fig. S1** The hydrodynamic diameter of GONs was detected by dynamic light scattering.

**Fig. S2** Cytotoxicity and cellular uptake of GONs.

**Fig. S3** Original images of the relative cellular fluorescence intensity analyzed using ImageJ software.

**Fig. S4** Flow cytometry images of cell cycle distribution in the three studied cell lines at 24 h after radiation.

**Fig. S5** Flow cytometry images of the apoptotic rates. **a** The fluorescence images of single A549 cell detected by Amnis flow cytometry. **b, c** Flow cytometry images of the apoptotic rates of NH1299 (b) and NH1650 (c) cells at 48 h after carbon ion radiation.

**Fig. S6** Flow cytometry images of autophagic rates at 12 h after radiation in A549 (a, d), NH1299 (b, e) and NH1650 (c, f) cells subjected to various treatments in the absence or presence of 3-MA.

**Supplementary figures**

**
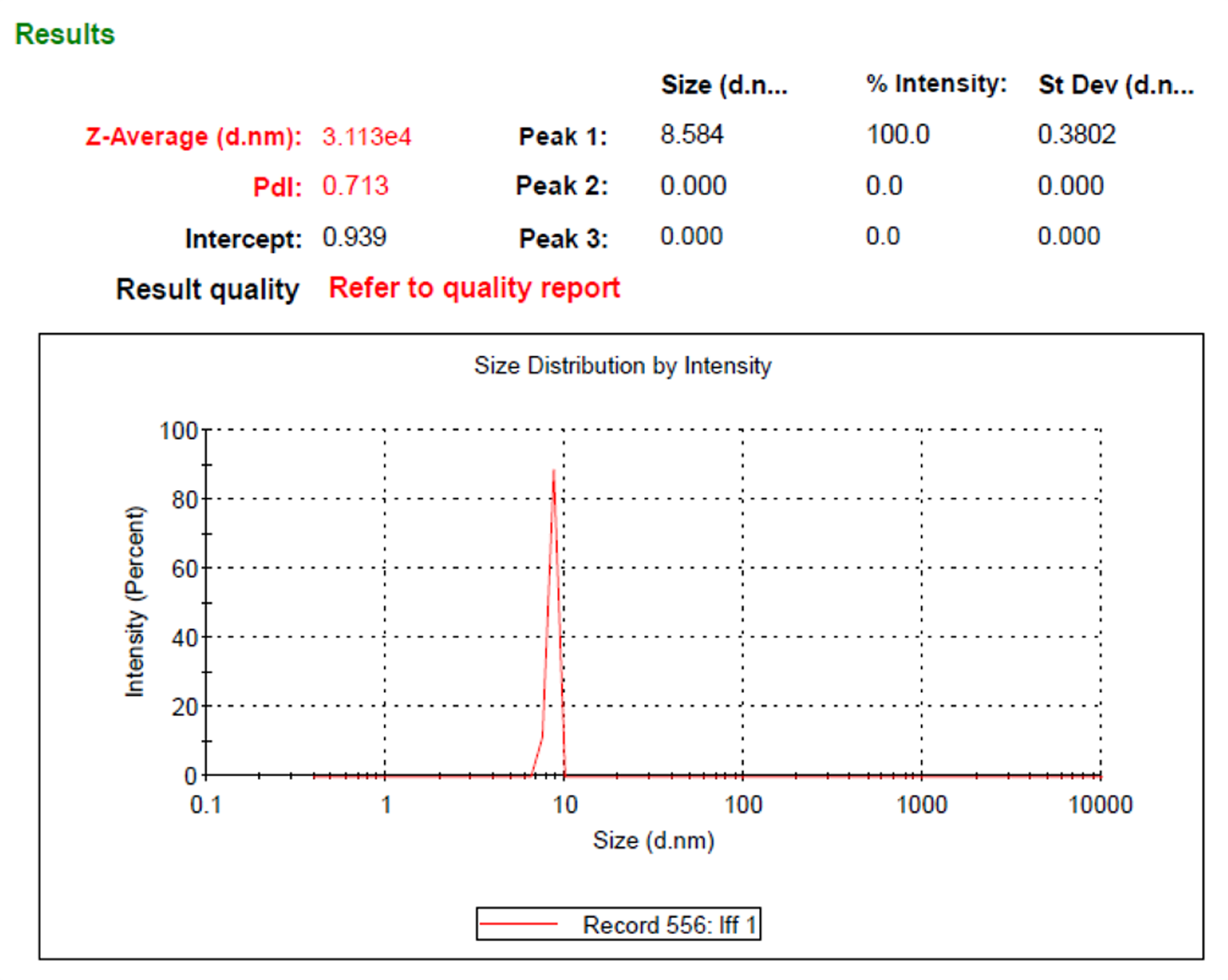
**

**Fig. S1** The hydrodynamic diameter of GONs was detected by dynamic light scattering.

**
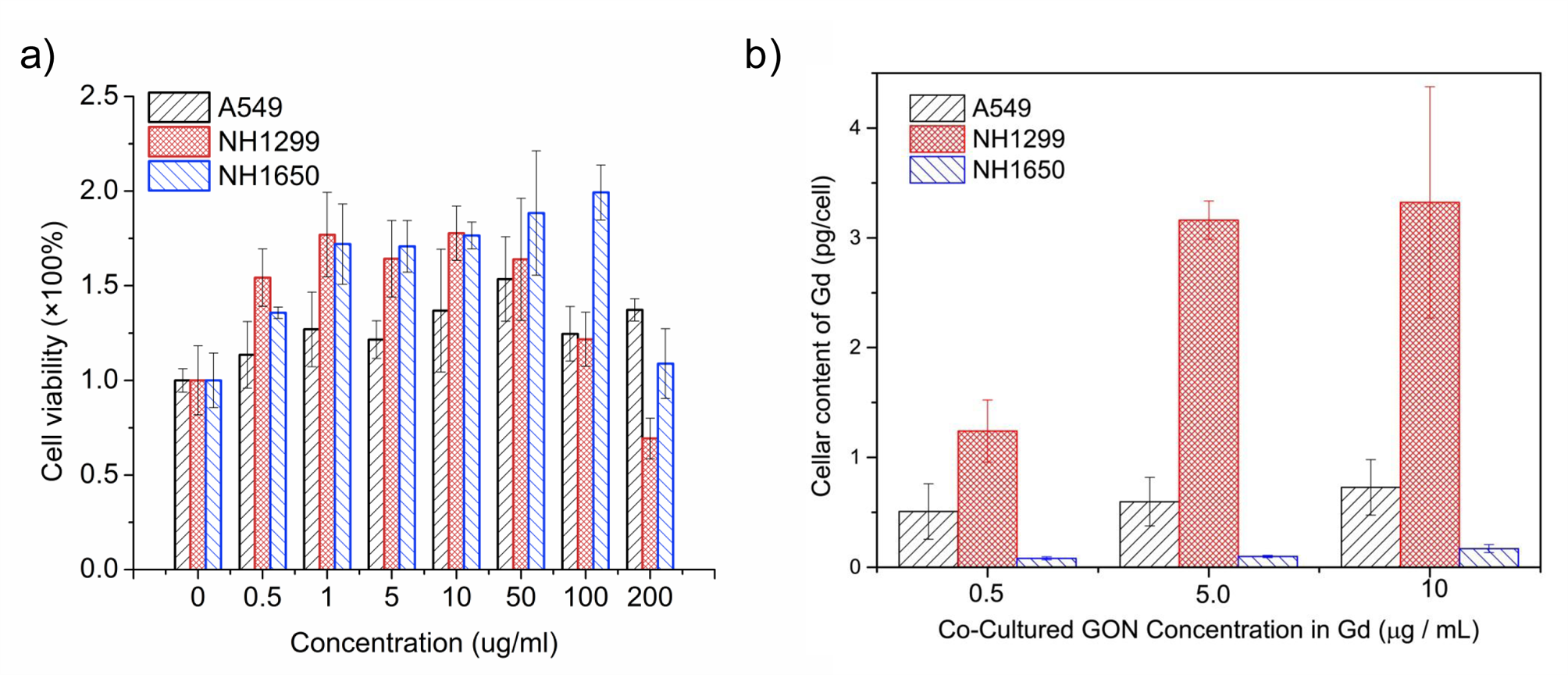
**

**Fig. S2** Cytotoxicity and cellular uptake of GONs.

**
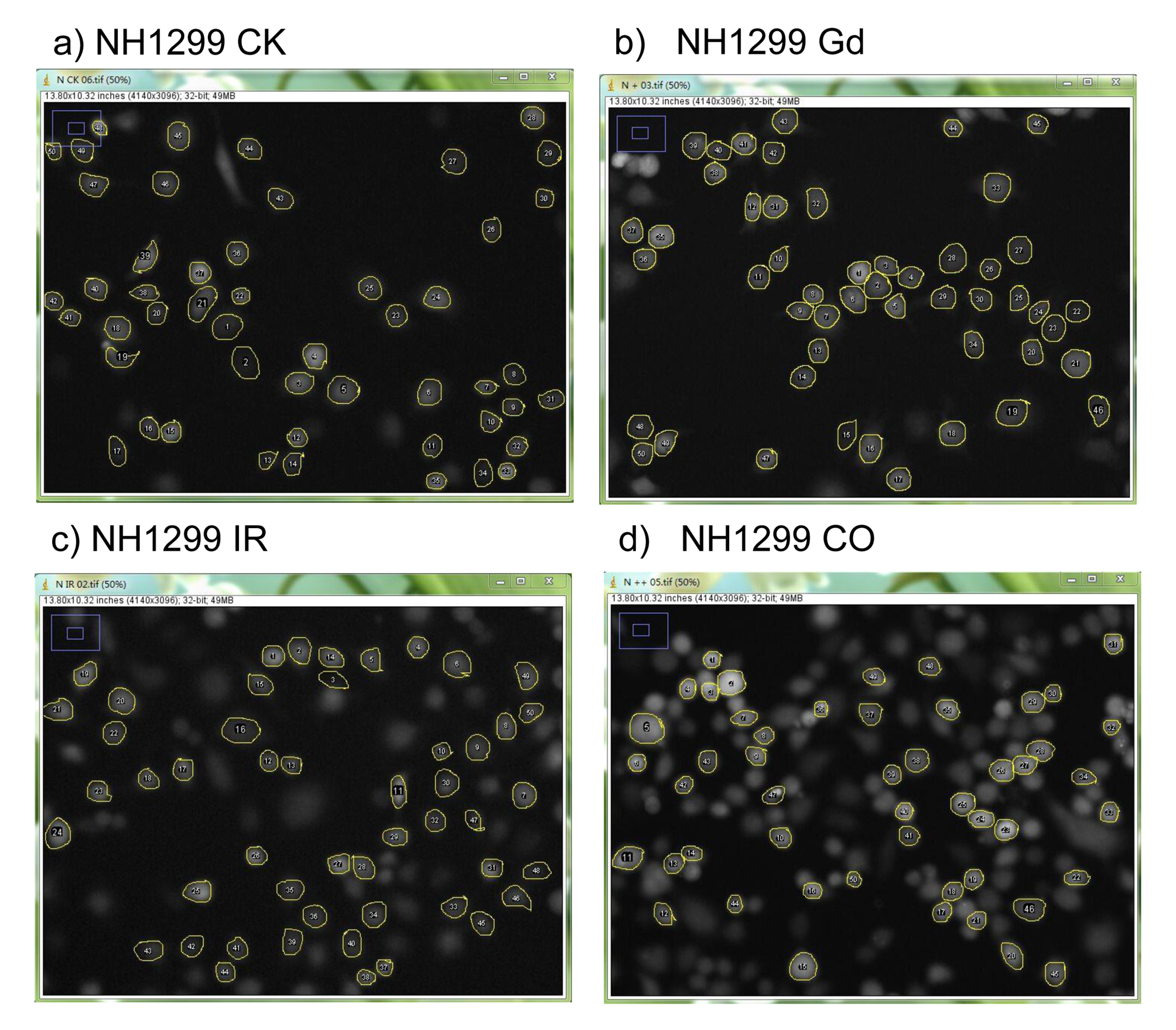
**

**Fig. S3** Original images of the relative cellular fluorescence intensity analyzed using ImageJ software.

**
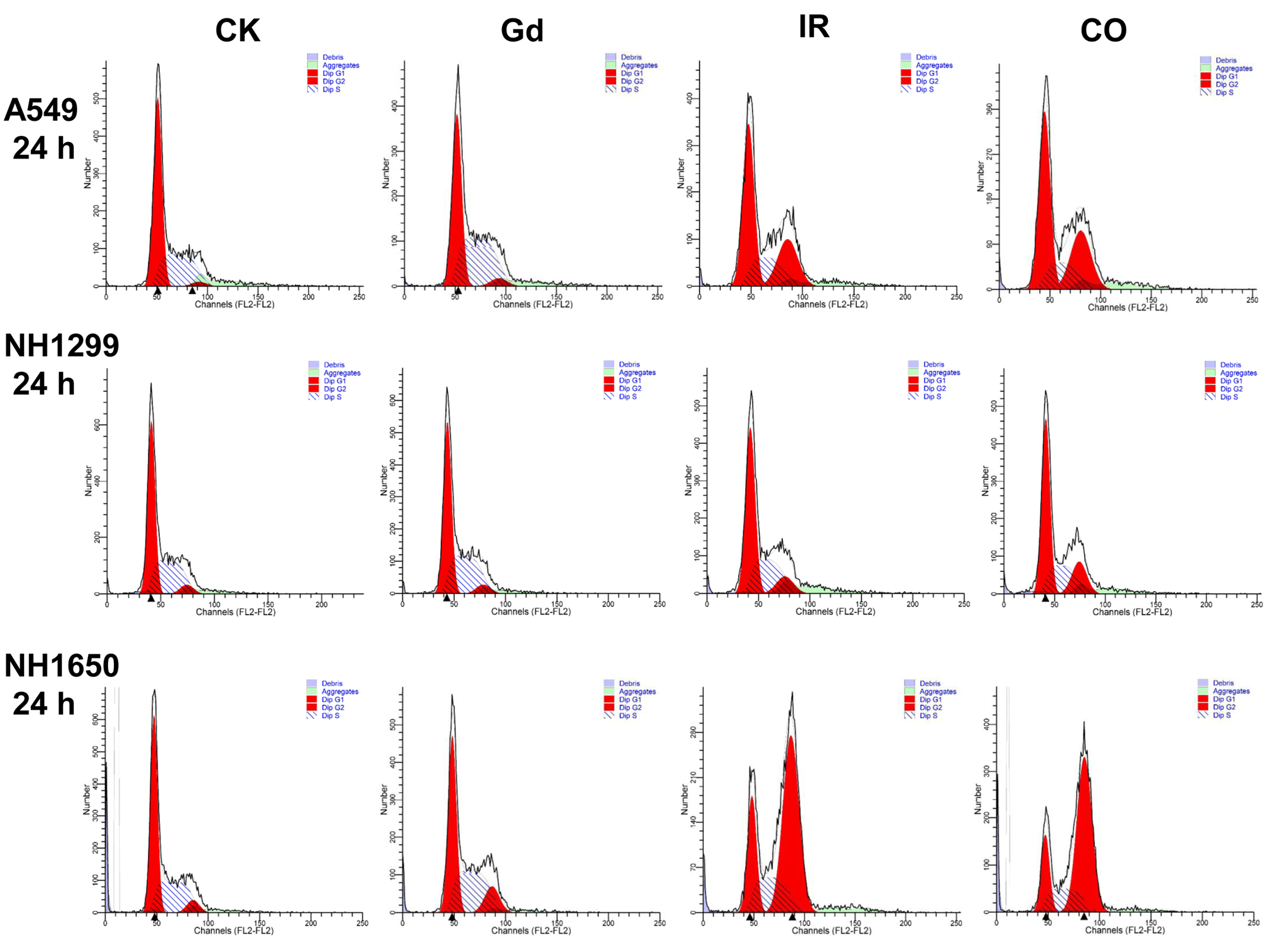
**

**Fig. S4** Flow cytometry images of cell cycle distribution in the three studied cell lines at 24 h after radiation.

**
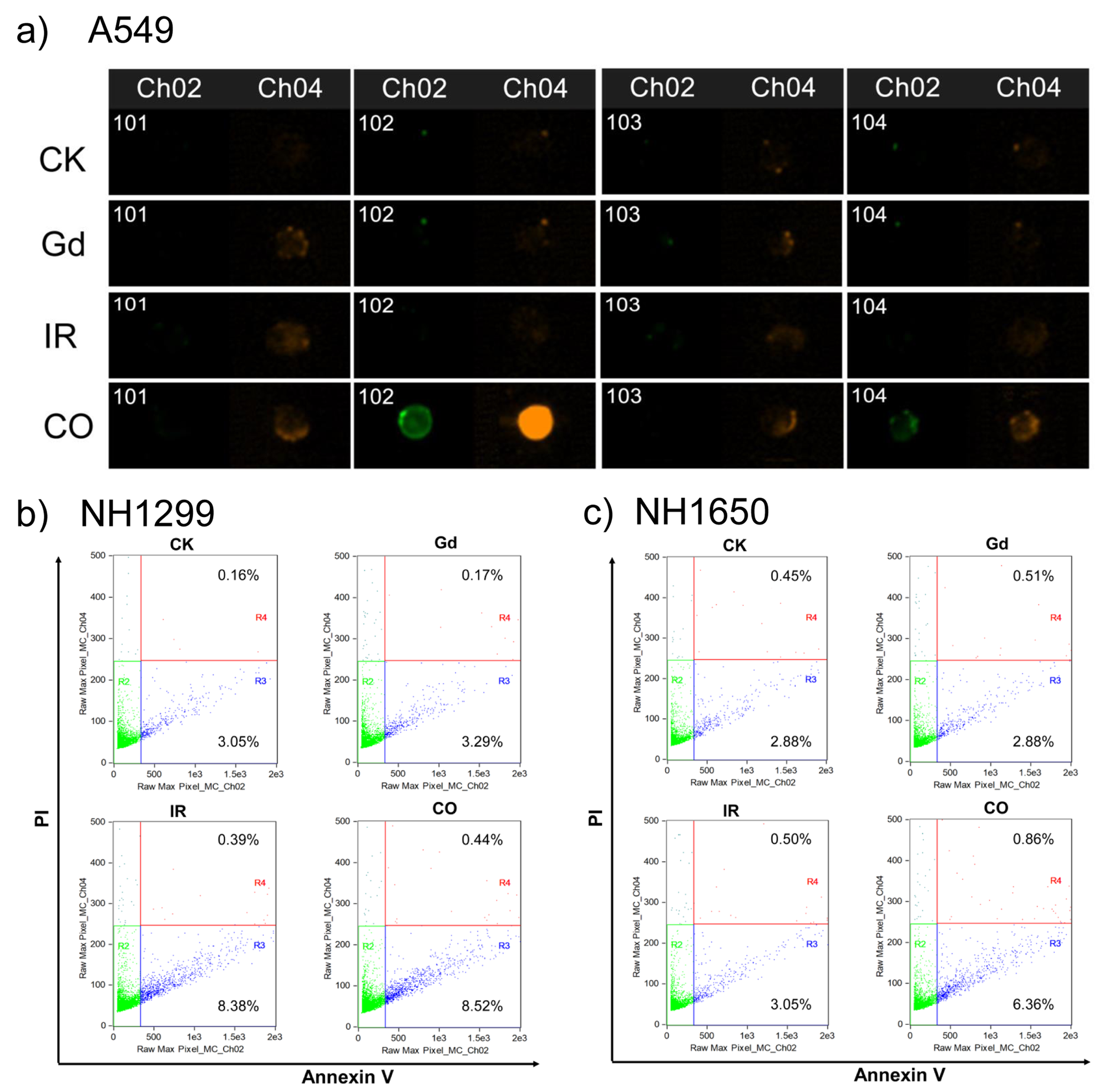
**

**Fig. S5** Flow cytometry images of the apoptotic rates. **a** The fluorescence images of single A549 cell detected by Amnis flow cytometry. **b, c** Flow cytometry images of the apoptotic rates of NH1299 (b) and NH1650 (c) cells at 48 h after carbon ion radiation.

**
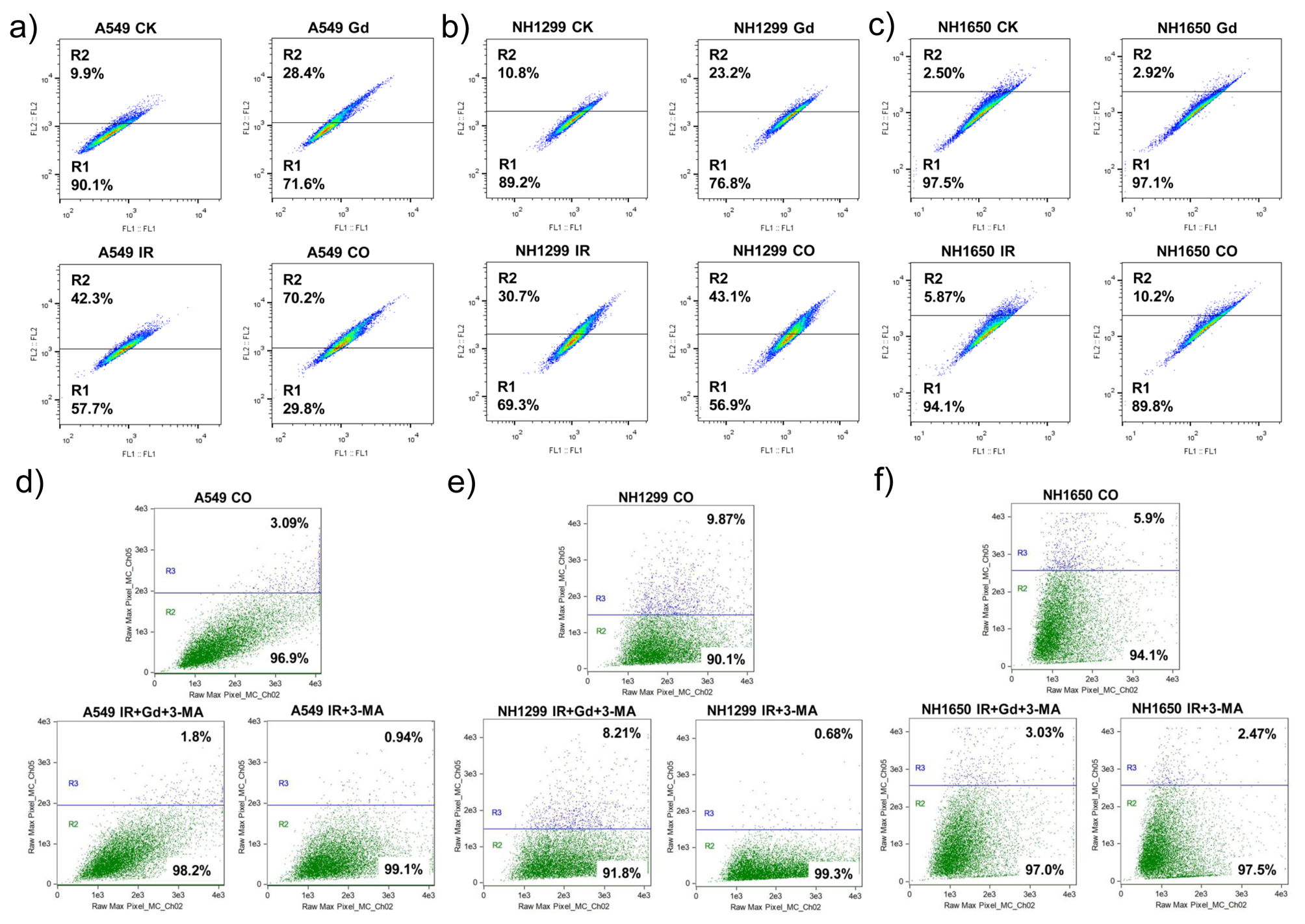
**

**Fig. S6** Flow cytometry images of autophagic rates at 12 h after radiation in A549 (a, d), NH1299 (b, e) and NH1650 (c, f) cells subjected to various treatments in the absence or presence of 3-MA.

**References**

[1] Soderlind F, Pedersen H, Petoral RM, Kall PO, Uvdal K. Synthesis and characterisation of Gd2O3 nanocrystals functionalized by organic acids. J. Colloid Interface Sci., 2005; 288: 140–148.

[2] Liu Y, Liu X, Jin X, et al. The dependence of radiation enhancement effect on the concentration of gold nanoparticles exposed to low- and high-LET radiations. Phys. Med., 2015; 31(3): 210–218.

[3] Dutreix J, Wanberie A, and Tubiana M, Introduction to Radiobiology, Taylor and Francis Ltd: London, UK, 1990.
